# The Molecular Interplay Between *Helicobacter pylori* Infection and Ulcerative Colitis: Unraveling Shared Biomarkers and Pathway Networks

**DOI:** 10.1002/iid3.70405

**Published:** 2026-03-17

**Authors:** Min Zhu, Xiao Li, Xinyuan Huang, Ruihua Dong, Shutian Zhang

**Affiliations:** ^1^ Department of Gastroenterology, Beijing Friendship Hospital Capital Medical University Beijing China; ^2^ State Key Laboratory of Digestive Health Beijing China; ^3^ National Clinical Research Center for Digestive Disease Beijing China; ^4^ Beijing Key Laboratory of Early Gastrointestinal Cancer Medicine and Medical Devices Beijing China; ^5^ Department of Research Ward, Beijing Friendship Hospital Capital Medical University Beijing China

**Keywords:** bioinformatics, biomarkers, *Helicobacter pylori*, immunity, inflammation, ulcerative colitis

## Abstract

**Background:**

Ulcerative colitis (UC) and *Helicobacter pylori* (*H. pylori*) infection show an intriguing inverse epidemiological association, but the underlying molecular mechanisms remain unclear, with emerging evidence suggesting *H. pylori may* modulate colonic inflammation via systemic immune regulation.

**Methods:**

We used bioinformatics approaches, including gene set enrichment analysis (GSEA), differential expression analysis, functional enrichment (GO/KEGG), protein‐protein interaction (PPI) networks, upstream regulatory molecule prediction, and immune infiltration characterization, to analyze gene expression datasets from UC and *H. pylori‐*infected samples, aiming to identify the interconnections and regulatory networks between these two conditions.

**Results:**

GSEA identified 53 shared pathways, primarily innate immune response pathways (e.g., TLR/NLR signaling, NF‐κB/IL‐17 cascades). We found 243 co‐differentially expressed genes enriched in leukocyte chemotaxis, cytokine activity, and extracellular matrix organization. Six hub genes (CXCL8, IL1B, MMP9, CXCL1, IFNG, CXCL9) were validated as robust diagnostic markers (AUC > 0.815 for both conditions). Immune landscape analysis revealed pan‐infiltration of immune cells in *H. pylori*‐infected tissues and shared dysregulated immune cells in UC tissues, with hub genes positively correlated with immune cell infiltration in both. We also identified regulatory miRNAs (e.g., miR‐204‐5p) and transcription factors (FOXC1, YY1) modulating these hub genes.

**Conclusion:**

This study uncovers shared immune‐mediated pathways and hub genes linking *H. pylori* infection to UC, establishing a molecular framework. These hub genes and regulatory networks may serve as diagnostic biomarkers and therapeutic targets, highlighting the need to investigate *H. pylori*‐driven immune modulation in UC pathogenesis.

**Abbreviations**
AUC: Area under the curve
BP: biological process
CC: cellular component
CD: Crohn's disease
CI: confidence interval
DEGs: differentially expressed genes
EPC: edge percolated component
FDR: false discovery rate
GEO: Gene Expression Omnibus
GO: Gene Ontology
GSEA: gene set enrichment analysis
*H. pylori*
: *Helicobacter pylori*
IBD: inflammatory bowel disease
KEGG: Kyoto Encyclopedia of Genes and Genomes
MCC: Maximum cross‐correlation
MF: Molecular function
miRNAs: MicroRNAs
MNC: maximum neighborhood component
OR: odds ratio
PPI: protein‐protein interaction
ROC: receiver operating characteristic
ssGSEA: single‐sample Gene Set Enrichment Analysis
TFs: transcription factors
UC: ulcerative colitis

## 1 Introduction

Ulcerative colitis (UC), a chronic and relapsing inflammatory disease characterized by intestinal inflammation and epithelial damage, affects a significant number of patients. Globally, UC incidence continues to rise, posing formidable challenges to global public health [1, 2]. The pathogenesis of UC involves multifactorial mechanisms including genetic susceptibility, environmental triggers, dysregulated immune responses, and altered host‐microbiota crosstalk [3, 4].

Although the global infection rate of *Helicobacter pylori* (*H. pylori*) has declined in recent years, it substantial at 43.1% worldwide, affecting billions of individuals [5]. Infection rates exhibit marked geographic disparity, with pronounced decreases in industrialized nations versus persistently high rates in developing regions. Notable, this epidemiological pattern inversely parallels the rising incidence of inflammatory bowel disease (IBD) in industrialized countries [2]. An umbrella review quantified this relationship, showing that *H. pylori*‐infected individuals have a 47% lower risk of UC (odds ratio [OR] = 0.53, 95% confidence interval [CI]: 0.44–0.65), with stronger protection observed in children than adults (OR, 0.24 vs 0.46) [6]. Such findings align with mounting epidemiological evidence supporting an inverse correlation between gastric *H. pylori* colonization and IBD incidence [7, 8]. Critics have argued this negative association might stem from confounding by hygiene‐related factors rather than direct biological effects [9]. However, extensive meta‐analyses consistently support the protective relationship, indicating that the reduced IBD risk associates with *H. pylori* infection is likely attributable to intrinsic properties of this gastric pathogen [10]. Mechanistically, *H. pylori* may inhibit IBD pathogenesis by inducing systemic immune tolerance and attenuating inflammatory responses [11]. Supporting this, studies have documented cases where *H. pylori* eradication therapy precipitated IBD onset [12], further reinforcing a causal link. Collectively, these observations suggest that gastric colonization by *H. pylori* may confers protection against IBD through specialized mechanisms. Nevertheless, the biological basis underlying this inverse association remains elusive, and the core molecular mediators linking gastric *H. pylori* infection to colonic inflammation remain have yet to fully explored.

While previous studies have sought to characterize the biomarker profiles associated with IBD and *H. pylori* infection, this project narrows its focus to the shared characteristics between UC and *H. pylori* infection in rigorously selected patient cohorts. This targeted approach is justified by the well‐documented differences between UC and Crohn's disease (CD) in both biological characteristics and pathogenesis [13]. To explore these shared characteristics, we employed comprehensive bioinformatics approaches to analyze gene expression datasets from *H. pylori*‐infected and UC samples. Through integrated analyses of differentially expressed genes (DEGs), functional enrichment (including Gene Ontology (GO) and Kyoto Encyclopedia of Genes and Genomes (KEGG) analysis), hub gene identification, immune infiltration profiling, and upstream regulatory molecules identification, we aimed to identify shared dysregulated biomarkers and pathways, which could shed light on the interplay between *H. pylori* infection and UC pathogenesis.

## 2 Materials and Methods

### 2.1 Data Acquisition and Preprocessing

**Figure 1:**
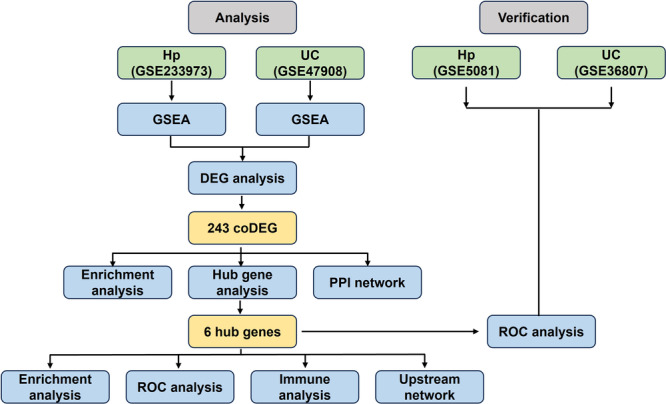
Flow chart of research design.

Gene expression data were retrieved from the Gene Expression Omnibus (GEO) database (http://www.ncbi.nlm.nih.gov/geo/), with selection criteria detailed in Figure 1. Four microarray datasets were analyzed: UC datasets (GSE47908, GSE36807) and *H. pylori* datasets (GSE233973, GSE5081). The UC datasets and GSE5081 (*H. pylori*) utilized the GPL570 platform ([HG‐U133_Plus_2] Affymetrix Human Genome U133 Plus 2.0 Array), while GSE233973 (*H. pylori*) employed GPL21185 (Agilent‐072363 SurePrint G3 Human GE v3 Microarray). The UC cohort comprised 19 patients/15 controls (GSE47908, test set) and 15 patients/7 controls (GSE36807, validation set). The *H. pylori* cohort included 13 infected/9 control samples (GSE233973, test set) and 16 infected/8 controls (GSE5081, validation set). All analyses were performed using R (version 4.3.2). All datasets underwent uniform preprocessing methods: 1) Background correction and normalization: Raw probe intensity data were imported and background correction was performed using the rma method via the backgroundCorrect function in the limma package (v3.58.1). Subsequently, quantile normalization was applied across all arrays using the normalizeBetweenArrays function to eliminate technical variation.; 2) Probe‐to‐gene annotation: Platform‐specific annotation files (GPL) were programmatically retrieved from the NCBI GEO database using the getGEO function from the GEOquery package (v2.70.0). The probe IDs in the normalized expression matrix were then mapped to official gene symbols in the GPL annotation table. Probes that lacked a corresponding gene symbol or mapped to non‐specific identifiers were flagged for removal.; 3) Expression matrix consolidation: The annotated expression matrix was cleaned by removing all probes without a valid, unique gene symbol. For genes measured by multiple probes, expression values were aggregated to a single representative value per gene per sample by calculating the arithmetic mean. This step ensured the final matrix contained unique gene‐level expression profiles and was performed using base R's aggregate function.

### 2.2 Gene Set Enrichment Analysis (GSEA)

GSEA was conducted using the clusterProfiler package (v4.10.1) in R (v4.3.2). A pre‐ranked list of genes was generated by sorting genes according to log2(fold‐change) values from differential expression analysis. The GSEA algorithm implemented the gseKEGG function for KEGG pathway analysis, performing 1000 permutations for statistical significance testing. Significantly enriched pathways were defined by a false discovery rate (FDR) < 0.05. Result visualization was performed using the enrichplot package (v1.22.0), with key findings presented as GSEA enrichment plots.

### 2.3 DEGs Identification

DEGs were identified for each discovery cohort using the limma package (v3.58.1). For the differential expression analysis, the following criteria were applied: absolute log₂(fold change) > 1.0 and adjusted p‐value (using the Benjamini‐Hochberg method) < 0.05. Overlapping DEGs between *H. pylori* and UC were defined as genes showing consistent dysregulation directions (upregulated or downregulated) in both datasets, which was determined using the VennDiagram package (v4.10.1).

### 2.4 Functional Enrichment Analysis

Overlapping DEGs were subjected to functional annotation via GO functional analysis and KEGG pathway enrichment analysis, implemented using the “clusterProfiler” package (v4.10.1). The GO classification encompasses three categories: biological process (BP), cellular component (CC), and molecular function (MF). For both GO and KEGG analyses, a *p*‐value < 0.05 was regarded as statistically significant. Functional enrichment analyses (GO/KEGG) of the top hub genes were conducted using the same method as described above. For GO analysis, we selected the top 10 significantly enriched terms within each of the BP, CC, and MF categories, along with the top 10 biologically relevant KEGG pathways, for visualization.

### 2.5 Construction of Protein‐Protein Interaction (PPI) Network and Hub Genes Selection

Overlapping DEGs were mapped to the STRING database (v12.0; https://string-db.org/) to explore molecular interactions patterns. A PPI network encompassing 243 common DEGs (co‐DEGs) associated with both *H. pylori* and UC was constructed, with the confidence score threshold set to > 0.4. This network was visualized using Cytoscape (v3.9.1) to identify regulatory genes, and the CytoHubba plugin in Cytoscape was employed, utilizing four distinct algorithms: maximum cross‐correlation (MCC), maximum neighborhood component (MNC), Degree, and edge percolated component (EPC). The top 10 key genes prioritized by each algorithm were analyzed, and hub genes were defined as those intersecting across all four sets of results.

### 2.6 Verification of Hub Genes

To validate the expression patterns and diagnostic utility of the six identified hub genes, a multi‐stage analytical approach was employed. First, the expression levels of these hub genes in the test dataset were rigorously assessed for their ability to discriminate between relevant phenotypes. This evaluation utilized receiver operating characteristic (ROC) curve analysis, implemented via the pROC package (v1.18.5) in R. For each hub gene, ROC curves were generated, and the area under the curve (AUC) was calculated to quantify its diagnostic performance. Subsequently, the expression patterns of these six hub genes were further validated in two independent validation datasets using limma (v3.58.1) to confirm their consistency and biological relevance across distinct cohorts. Finally, to evaluate the integrated diagnostic power of the six identified hub genes, a combined diagnostic model was built using binary logistic regression. The combined model's ability to discriminate between cases and controls was assessed by ROC curves and AUC along with its 95% confidence interval (CI) using the pROC package (v1.18.5). All ROC curves, including those for individual genes and the combined model, were visualized using the ggplot2 package (version 3.5.2).

### 2.7 Immune Cell Infiltration Analysis

Single‐sample Gene Set Enrichment Analysis (ssGSEA) was used to quantify the level of immune cell infiltration. Immune gene signatures were curated from published metagene resources (Supplementary Table 1), containing 28 immune cell‐type‐specific metagenes. These gene sets were structured as a GeneSetCollection object using GSEABase (v1.64.0). ssGSEA was implemented through GSVA package (v1.50.5) with Gaussian kernel density estimation. Enrichment scores were standardized for each cell type using Z‐score transformation to facilitate cross‐sample comparison. The top 16 most variable immune cell subtypes which ranked by inter‐sample variance, were retained for downstream visualization. Clustered heatmaps were generated using the pheatmap package (v1.0.12). For comparative analysis of immune infiltration across clinical groups, Wilcoxon rank‐sum tests were applied to the enrichment scores of each immune cell type, with FDR adjustment via Benjamini‐Hochberg procedure. Boxplots were generated with the ggplot2 package (v3.5.2).

### 2.8 Construction of microRNAs (miRNAs) and Transcription Factors (TFs) Regulatory Network of the Hub Genes

The miRNA‐target gene interactions and TF‐ target gene network were constructed and visualized using NetworkAnalyst (https://www.networkanalyst.ca/NetworkAnalyst/) [14]. Specifically, the 6 hub genes (using their gene symbols) were directly uploaded to the platform; the JASPAR database was then used to predict their interacting TFs, while the miRTarBase (v9.0) database was employed for miRNAs target prediction.

## 3 Results

### 3.1 GSEA Analysis in *H. pylori* Infection and UC

**Figure 2:**
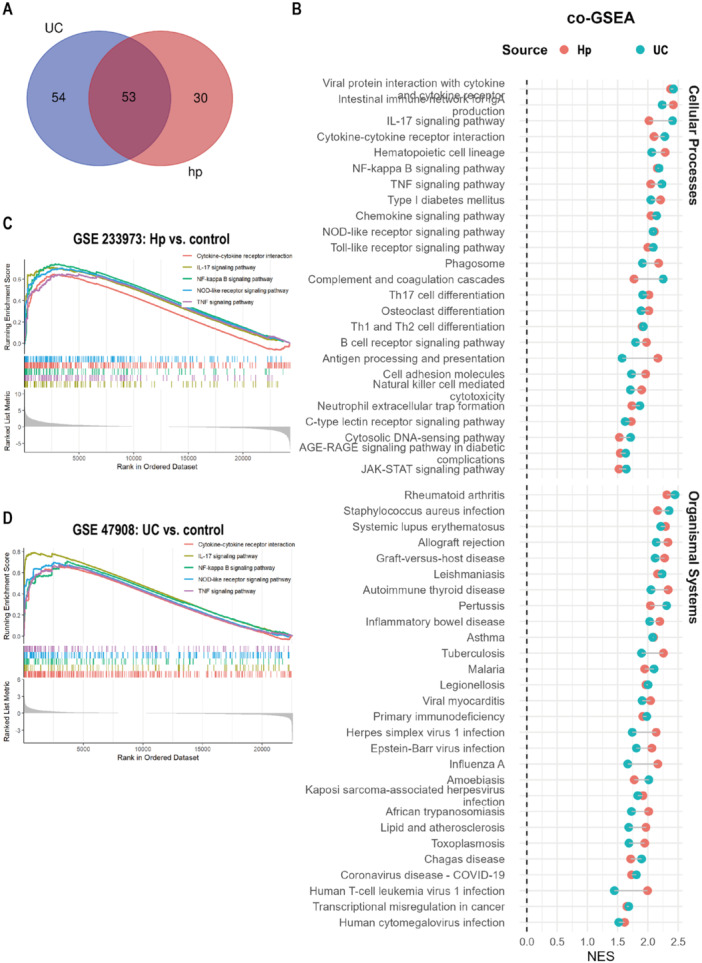
GSEA analysis for GSE47908 (UC) and GSE233973 (H. pylori). (A) Venne plot showed the number of significant pathways shared in both conditions. (B) Significant pathways enrich in both diseases. (C‐ D) Immune‐related pathways were significantly enriched in both UC and H. pylori infection.

GSEA identified 107 enriched pathways in UC and 83 in *H. pylori* infection, with 53 pathways shared between the two conditions (Figure 2A). Among these overlapping KEGG pathways, cellular processes and organismal systems accounted for 47.2% (25 pathways) and 52.8% (28 pathways), respectively (Figure 2B). Within the cellular processes category, the intersecting pathways were predominantly associated with the immune system. Specifically, immune‐related pathways were significantly enriched in both UC and *H. pylori* infection, with a particular emphasis on those involved in innate immune response activation, including the Toll‐like receptor signaling, NOD‐like receptor signaling, and C‐type lectin receptor signaling pathways (Figure 2B). Notably, NF‐κB‐mediated inflammation, TNF signaling pathway, as well as IL‐17 signaling pathways, ranked among the top overlapping signatures enriched in both conditions (Figure 2C,D).

### 3.2 Identification of DEGs in *H. pylori* Infection and UC

**Figure 3:**
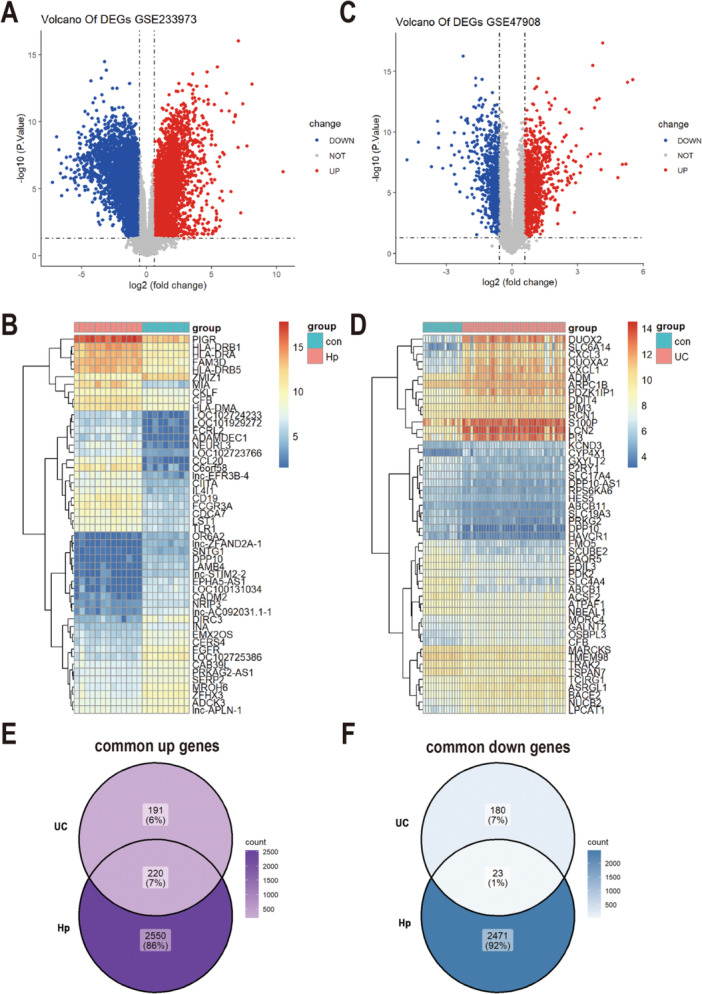
Co‐DEGs in *H. pylori* and UC. (A) Volcano plots of DEGs from GSE233973. (B) Volcano plots of DEGs from GSE47908. (C) The heatmap plot of Top 25 DEGs from GSE233973, red and blue grids indicate up‐and down‐regulated DEGs, respectively. (D) The heatmap plot of Top 25 DEGs from GSE47908. (E, F) Venn diagram showed the intersection of common upregulated (E) and common downregulated (F) DEGs in two diseases. DEGs, differentially expressed genes.

As depicted in Figure 3A,B, a total of 5,264 DEGs were identified between the *H. pylori*‐infected and control groups, with 2,770 being upregulated and 2,494 downregulated. For UC, 614 DEGs were detected in UC tissues compared to healthy controls (411 upregulated and 203 downregulated using filtering criteria of |fold change | > 1 and adjusted *p* < 0.05) (Figure 3C,D). Furthermore, 243 common DEGs (co‐DEGs) were shared between *H. pylori* infection and UC, comprising 220 upregulated and 23 downregulated co‐DEGs (Figure 3E,F).

### 3.3 Functional Annotation of co‐DEGs via GO and KEGG Enrichment Analyses

**Figure 4:**
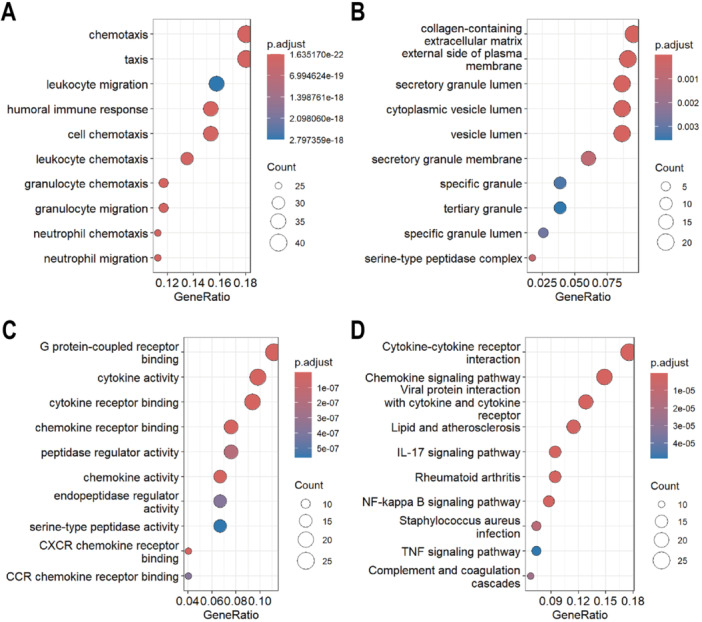
Functional enrichment analysis of co‐DEGs. (A) Biological process, (B) Cellular component and (C) Molecular function of GO analysis. (D) KEGG analysis. The size of the circle represents the number of genes enriched. DEGs, differentially expressed genes; GO, Gene Ontology; KEGG, Kyoto Encyclopedia of Genes and Genomes.

To functionally characterize the 243 identified co‐DEGs, we performed GO and KEGG pathway enrichment analyses. Notably, the majority of these enriched GO and KEGG terms were associated with immune‐related functions. Specifically, In the BP category, enrichment analysis highlighted genes involved in inflammatory cells chemotaxis and migration (e.g. leukocyte and granulocyte) (Figure 4A), underscoring their roles in the recruitment of inflammatory cells. For the CC category, the co‐DEGs are localized to extracellular matrix components and the lumen/membrane of secretory granules (Figure 4B), suggesting potential involvement in intercellular communication and vesicular transport. MF enrichment analysis predominantly identified cytokine and chemokine activity (Figure 4C), consistent with their predicted roles in immune signaling. KEGG pathway analysis further revealed enrichment in inflammatory cascades, including chemokine signaling, IL‐17, NF‐κB, and TNF pathways, as well as pathways related to infectious diseases (e.g., Staphylococcus aureus infection) (Figure 4D).

### 3.4 Construction of PPI Network of co‐DEGs and Acquisition of Key Gene Modules

**Figure 5:**
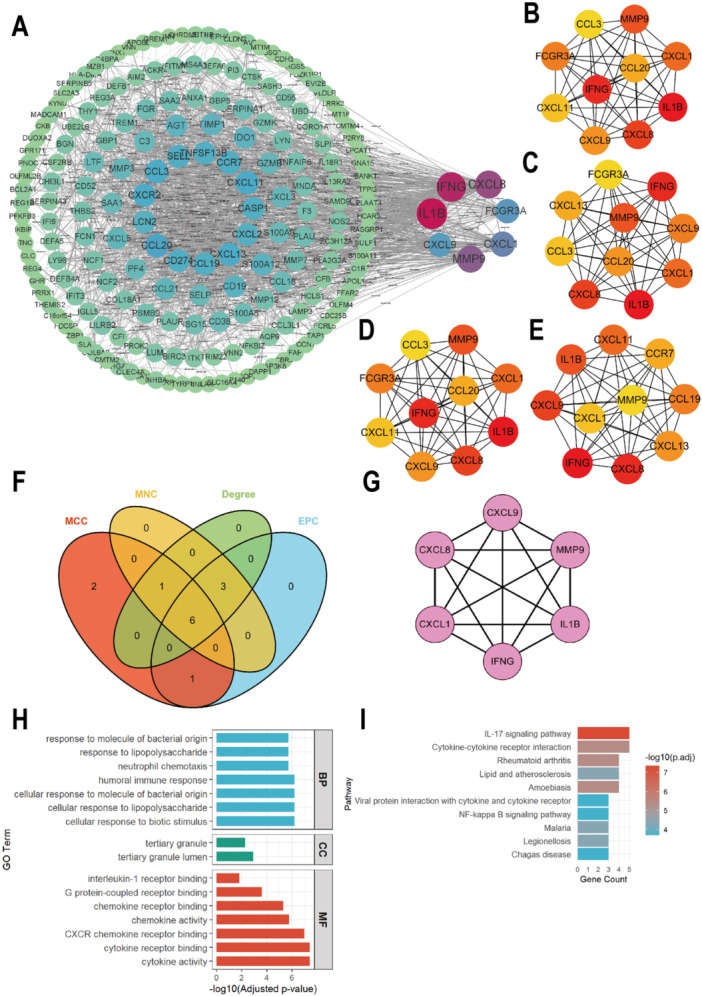
The PPI network of co‐DEGs. (A). The PPI network of interacted genes. (B‐E). Top 10 interacted genes were screened out by ‘MNC’(B), ‘EPC’(C), ‘Degree’(D), ‘MCC’(E) algorithm and visualized as a network in Cytoscape. (F) Venne plot showed the intersecting results of the four algorithms. (G) The network of identified 6 hub genes. (H) GO enrichment analyses of these hub genes. (I) KEGG enrichment analyses of these hub genes. DEGs, differentially expressed genes; PPI, protein‐protein interaction; GO, Gene Ontology; KEGG, Kyoto Encyclopedia of Genes and Genomes.

To characterize the PPI network of the 243 co‐DEGs identified above, we analyzed their interactions using the STRING database. The resultant PPI network consisted of 191 nodes and 1,641 edges, with 52 genes lacking reported functional annotations. In this network, node size was proportional to connectivity (degree), with higher connectivity indicating greater centrality. The top seven hub genes (IFNG, CXCL8, IL1B, MMP9, CXCL1, FCGR3A, CXCL9) were identified based on connectivity (Figure 5A). Using the cytoHubba plugin, four submodules were screened, and the top 10 proteins displayed (Figure 5B–E). Six core hub genes (CXCL8, CXCL9, MMP9, CXCL1, IFNG, IL1B) were identified by intersecting results from multiple algorithms (Figure 5F), and their interconnections were visualized in Figure 5G. GO and KEGG enrichment analyses were performed on these hub genes. BP analysis revealed significant enrichment in responses to bacterial molecular components, lipopolysaccharide, neutrophil chemotaxis, and the humoral immune response (Figure 5H). KEGG pathway analysis showed enrichment in inflammatory signaling pathways (IL‐17, NF‐κB) and infection‐related diseases (amoebiasis, malaria, leishmaniasis, Chagas disease) (Figure 5I).

### 3.5 Validation of Hub Genes as Diagnostic Biomarkers

**Figure 6:**
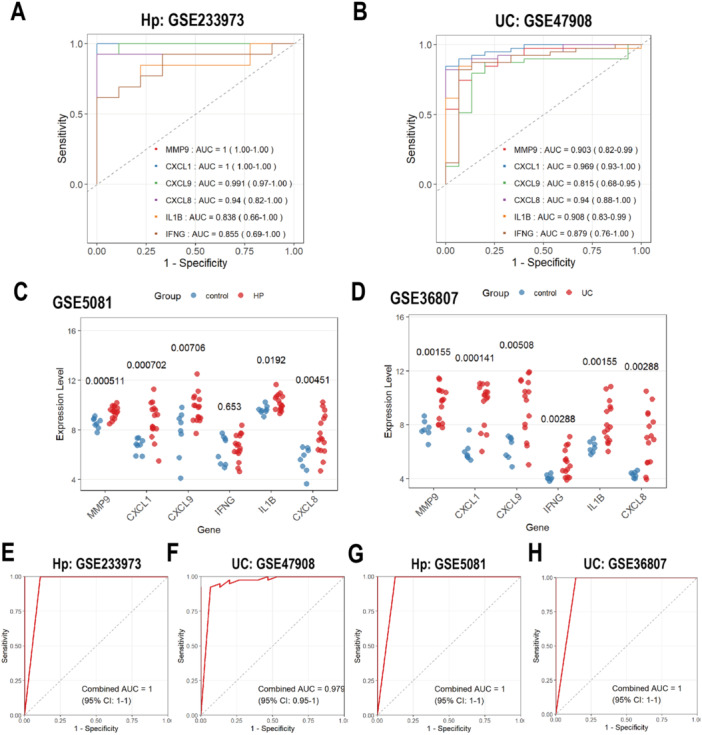
ROC analysis and validation of hub genes. (A) ROC curves of hub genes in *H. pylori* infection (GSE233973). (B) ROC curves of hub genes in UC (GSE47908). (C) Validation of expression levels in *H. pylori* infection (GSE5081). (D) Validation of expression levels in UC dataset (GSE36807). (E‐H) ROC curves of combination of the 6 genes in different datasets. ROC, receiver operating characteristic; AUC, area under the curve; UC, ulcerative colitis.

ROC analyses were performed to evaluate the diagnostic potential of the six hub genes in both *H. pylori* infection and UC. In *H. pylori* infection datasets, all six genes exhibited AUC values > 0.838 (Figure 6A), while in UC datasets, their AUCs exceeded 0.815 (Figure 6B), indicating strong diagnostic accuracy. To validate these findings, external datasets were used to assess the expression patterns of these hub genes. In an *H. pylori*‐specific external cohort, MMP9, CXCL1, CXCL9, and CXCL8 were significantly upregulated compared to healthy controls (Figure 6C), whereas IFNG expression showed no significant difference. Similarly, all 6 hub genes were markedly upregulated in UC patient samples relative to controls (Figure 6D, *p* < 0.01), reinforcing their diagnostic utility. Notably, combining the 6 genes dramatically improved diagnostic efficiency, with AUC values approaching 100% (Figure 6E–H). These results suggest that these hub genes may serve as highly robust diagnostic biomarkers for both conditions.

### 3.6 Immune Landscape Analysis

**Figure 7:**
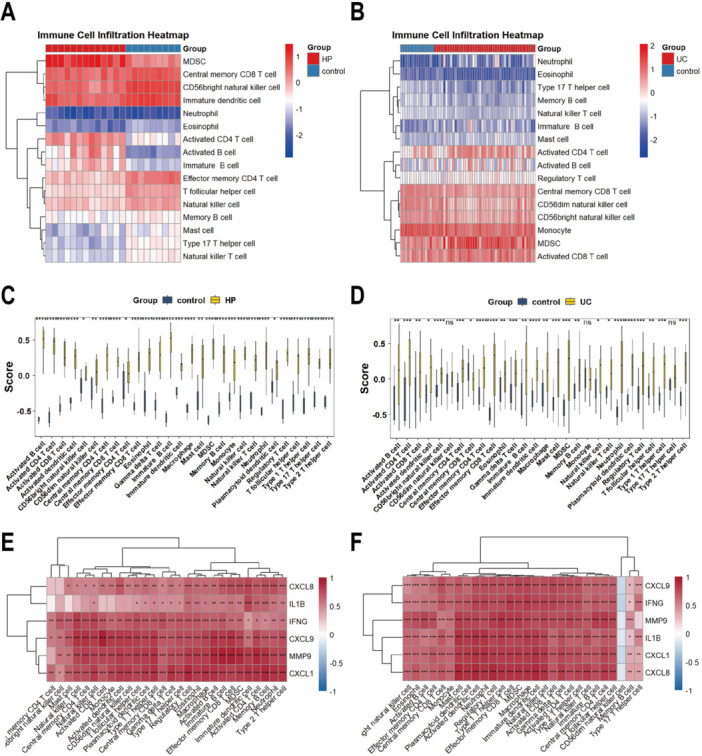
Immune landscape analysis. (A, B) Heatmap displayed the distribution of the relative infiltration levels of top16 immune cells picked out from 28 immune cells in GSE233973 (A) and GSE47908 (B). (C, D) The box plot comparing the infiltration levels of immune cells between disease and control group in GSE233973 (C) and GSE47908 (D). (E, F) The correlated heatmap between 6 key genes and immune cells in GSE233973 (E) and GSE47908 (F).

The ssGSEA algorithm was applied to quantify the relative infiltration levels of the top 16 immune cell types selected from a panel of 28 immune cell types (Figure 7A,B), along with their relative expression profiles (Figure 7C,D). Compared to the control group, *H. pylori*‐infected tissues exhibited increased infiltration of all 28 immune cell types (Figure 7C). In UC tissues, central memory CD8⁺ T cells were downregulated, while Th17 cells, monocytes, and CD56dim natural killer cells showed no statistically significant changes. All other immune cells were upregulated (Figure 7D). Correlation analysis between the six hub genes and the infiltration level of the 28 immune cell types revealed that, in *H. pylori*‐infected tissues, IL1B showed weak correlations with effector memory CD4 T cells, CD56bright natural killer cells, mast cells, natural killer T cells, central memory CD4 T cells, activated CD8 T cells, monocytes, active dendritic cells, and CD56dim natural killer cells. However, the other hub genes exhibited significant positive correlations with nearly all immune cell types, indicating a strong association between hub gene expression and immune cell infiltration (Figure 7E). In UC, except for CD56dim NK cells, infiltration of all other immune cell types showed a positive correlation with these hub genes (Figure 7F).

### 3.7 Prediction and Validation of Regulatory Molecules Targeting Hub Genes

**Figure 8:**
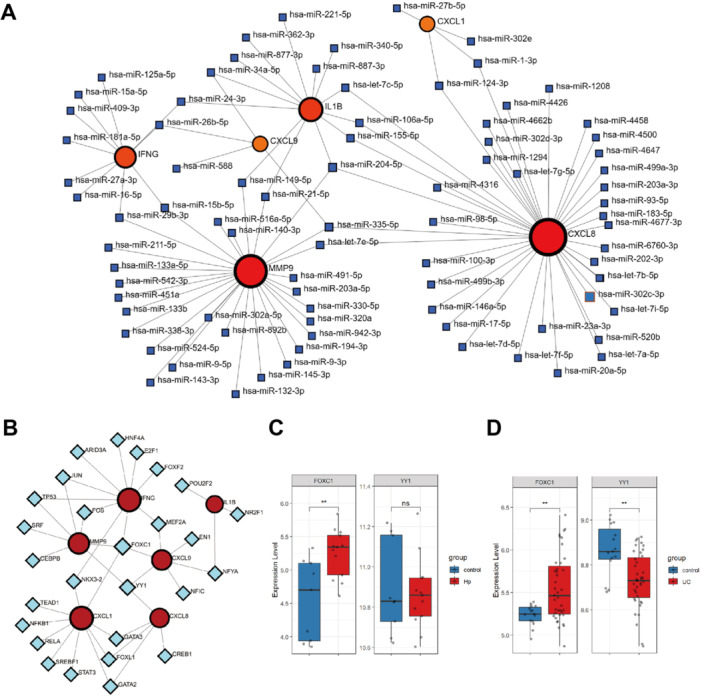
Prediction and validation of regulatory molecules targeting hub genes. (A) Regulatory network between predicted miRNAs and hub genes. (B) Regulatory network between transcription factors and hub genes. (C, D) The expression levels of FOXC1 and YY1 in *H. pylori*‐infected tissues (GSE233973) (C) and UC tissues (GSE47908) (D).

A total of 83 miRNAs targeting the hub genes were identified. Prediction results showed that 40 miRNAs may regulate CXCL8, 29 miRNAs may regulate MMP9, 13 miRNAs may regulate IL1B, 10 miRNAs may regulate IFNG, and 4 miRNAs may regulate CXCL9 and CXCL1, respectively. Notably, miR‐204‐5p and miR‐335‐5p were predicted to target IL1B, MMP9, and CXCL8 (Figure 8A). In addition, 27 TFs were identified, and a TF regulatory network was constructed to characterize their interactions with the hub genes (Figure 8B). The top two TFs, which interacted with at least three hub genes, were FOXC1 and YY1. Furthermore, the expression levels of FOXC1 and YY1 were validated: FOXC1 was significantly upregulated in both *H. pylori*‐infected and UC tissues, whereas YY1 was downregulated in UC tissues but exhibited no significant change in *H. pylori* infection (Figure 8C,D).

## 4 Discussion

This work represents a comprehensive integration of pathway analysis, gene co‐expression, and immune infiltration profiling to dissect the overlapping molecular signatures of *H. pylori* infection and UC. A key innovation lies in the identification of a conserved set of hub genes with dual diagnostic potential, which not only enhances our understanding of the immunopathological links between infectious and inflammatory gastrointestinal diseases, but also offers promising biomarkers for clinical diagnosis. By bridging these two distinct yet mechanistically related conditions, our findings may provide a theoretical foundation for developing targeted therapeutic strategies that leverage shared regulatory pathways.

Numerous epidemiological studies have consistently demonstrated an inverse association between *H. pylori* infection and IBDs, even after accounting for confounding factors such as age, ethnicity, diagnostic methods, and prior medication use [15, 16]. Notably, research spanning both pediatric and adult populations has linked *H. pylori* infection to reduced IBD severity [17], suggesting a potential protective role in mitigating disease onset or exacerbation. Clinical data further support this trend: compared to UC patients without *H. pylori* infection, those with *H. pylori* infection exhibited lower mortality rate and reduced hospital costs, despite comparable lengths of stay. Although not statistically significant, these patients also showed diminished rates of intestinal perforation and intra‐abdominal abscess formation, indicating that *H. pylori* may modulate the progression of UC through underlying mechanisms [18].

Despite these observed associations, the protective mechanism by which *H. pylori* colonization in the upper digestive tract confers protection against IBD remain poorly understood, and the molecular interplay governing the relationship between *H. pylori* and UC remains largely elusive. The unresolved causal relationship between *H. pylori* and UC directly impacts clinical decision‐making, fueling ongoing debates over whether eradication therapy is warranted for all infections. Identifying factors and mechanisms that link *H. pylori* infection with a reduced incidence of UC, or even improved outcomes of UC patients, may be key to unraveling this causal enigma.

Several hypotheses have been proposed to elucidate how *H. pylori* modulates the host immune response to influence UC outcomes. First, *H. pylori* may alter the intestinal microbiota composition, thereby reshaping the local intestinal environment and immune response profiles, which could confer protection against IBD development [19, 20]. Second, *H. pylori*‐induced gastric inflammation triggers systemic cytokine release, leading to downregulation of the immune response and dampening of autoimmune processes [21]. This is supported by preclinical evidence: in multiple mouse models of IBD, live *H. pylori* infection or treatment with its immunomodulatory components significantly reduced clinical and histopathological severity. Proposed mechanisms include tolerization of dendritic cells, production of anti‐inflammatory cytokines, and preferential induction of regulatory T cells [22]. For instance, *H. pylori* infection alleviates dextran sodium sulfate‐induced colitis in mice, with CD19^+^IL‐10^+^ regulatory B cells appearing to mediate this protective effect, highlighting *H. pylori*'s ability to modulate intestinal mucosal immunity beyond the gastric niche [23]. Additionally, *H. pylori* activates Toll‐like receptors (TLRs) on dendritic cells (DCs), which can polarize T cell responses: DC‐derived IL‐12 promotes pro‐inflammatory Th1 responses, whereas IL‐10 drives anti‐inflammatory Th2/Treg differentiation [24]. Collectively, these pathways may underpin the observed protective effects of *H. pylori* against UC.

However, existing research has significant limitations: Critical knowledge gaps include the lack of cross‐tissue transcriptomic analyses comparing *H. pylori*‐infected gastric mucosa and UC‐affected colonic tissues; an insufficient understanding of shared immune dysregulation pathways between these two conditions; and the absence of systematic identification of pivotal genes regulating the gut‐stomach axis. To address these shortcomings, we performed a comprehensive bioinformatics analysis of gene expression datasets from *H. pylori*‐infected and UC samples. By integrating differential gene expression analysis, functional enrichment analyses (GO/KEGG), hub gene identification, immune infiltration profiling, and upstream regulatory molecules prediction, our study aimed to identify potential diagnostic biomarkers and therapeutic targets, thereby clarifying the molecular mechanisms underlying the complex relationship between *H. pylori* infection and UC pathogenesis.

The identified hub genes (CXCL8, CXCL9, MMP9, CXCL1, IFNG, IL1B) acted as critical molecular nodes linking *H. pylori*‐induced gastric inflammation and UC pathogenesis. For instance, CXCL8 (IL‐8) drove neutrophil chemotaxis through NF‐κB activation, which is a hallmark of inflammatory responses in both gastric and colonic tissues [25, 26]. IL1B induced CXCL9 secretion in gastric epithelial cells, potentially enhancing gastric mucosal defense [27]; conversely, elevated IL1B levels in intestinal tissues from UC patients and colitic mice reduced enterocyte occludin expression, thereby increasing epithelial tight junction permeability [28]. MMP9 expression was markedly upregulated in *H. pylori*‐infected tissues, where it functioned as a key mediator of neuroinflammatory responses and extracellular matrix remodeling [29]. Additionally, MMP‐9 levels correlated with disease severity in pediatric UC, associating with the Mayo score, Paris classification and C‐reactive protein levels [30]. CXCL1 and IFNG were highly expressed in *H. pylori*‐infected gastric tissues, emphasizing their roles in pathogen‐driven inflammation [31, 32]. In UC, elevated CXCL1 regulated macrophage polarization by modulating the Th1 cytokine IFN‐γ, thereby promoting colitis progression [33]. Collectively, the conserved upregulation of these genes in both conditions pointed to a “shared inflammatory hub”. Notably, chronic *H. pylori* infection triggered cytokine networks may paradoxically suppress colonic inflammation by priming systemic immune responses. This hypothesis was supported by the near‐universal positive correlation between hub genes and immune cell infiltration in *H. pylori* infection, indicating that these genes orchestrated a regulatory cascade capable of dampening excessive inflammation in distal tissues such as the colon.

Our study uncovered shared molecular signatures between *H. pylori* infection and UC, with immune dysregulation as a central theme. Through GSEA, 53 overlapping pathways were identified, and a critical intersection was found to be immune system activation, which includes the TLR/NLR signaling pathway and the NF‐κB/IL‐17 signaling pathway. Notably, *H. pylori*‐infected tissues and UC tissues exhibited similar pan‐immune cell infiltration patterns, and both conditions converged on innate immune response pathways, pointing to a conserved inflammatory circuitry. The enrichment of chemokine signaling and neutrophil chemotaxis further underscores shared mechanisms of immune cell recruitment. These findings aligned with prior evidence that *H. pylori* modulates systemic immunity. For instance, studies have shown that while *H. pylori* infection recruits substantial numbers of DCs to the gastric mucosa, these DCs displayed a functionally semi‐mature state with an immune‐tolerant phenotype [34, 35]. Furthermore, *H. pylori*‐stimulated DCs could promote regulatory T cell (Treg) differentiation to induce immune tolerance. This immune‐tolerant property of *H. pylori* may facilitate its persistent colonization of the gastric mucosa and enable it to exert systemic immunomodulatory effects on distant organs via lymphocyte recirculation, potentially influencing the pathogenesis of various autoimmune diseases, including UC [36]. This strongly suggests that they play a key role in driving a shared core inflammatory response. However, the long‐term functions of these molecules may differ between the chronic landscape of *H. pylori* infection and the chronic relapsing inflammation of UC. Specifically, whether they ultimately lead to tissue damage or participate in some form of immune regulation may require further clarification based on more specific cellular contexts and disease stages.

The identified hub genes exhibit high diagnostic accuracy, with AUC values surpassing 0.815 in both conditions, which highlights their potential as cross‐disease biomarkers. Notably, the combined panel of these genes achieved near‐perfect diagnostic efficiency (AUC ~ 100%) in our dataset, suggesting they could serve as robust markers for monitoring crosstalk between *H. pylori* infection and UC. However, this exceptional performance requires validation in larger, prospective, and multi‐center cohorts to assess its clinical utility and generalizability. Additionally, our identification of regulatory miRNAs (e.g., miR‐204‐5p, miR‐335‐5p) and transcription factors (FOXC1, YY1) that may modulate these hub genes provides novel insights into the upstream regulatory networks governing their expression. Furthermore, targeting conserved pathways such as IL‐17 and NF‐κB may offer dual therapeutic benefits for both *H. pylori*‐related gastritis and UC, though translational studies are warranted to validate this potential.

This investigation addresses a critical knowledge gap in the immunology of the “gut‐gastric axis”. However, our study has certain limitations. First, our analysis is solely based on in silico investigation of publicly available transcriptomic data, and it lacks experimental validation to confirm the causal or mechanistic roles of the identified hub genes and pathways in disease interplay. Second, the integration of data from multiple independent GEO datasets, though a strength in terms of sample size, introduces potential technical confounders. Despite applying stringent normalization within each dataset, residual batch effects arising from differences in sample collection, processing protocols, and platforms across the four cohorts cannot be fully ruled out. Third, although we utilized datasets from four distinct countries (Danish [GSE47908], British [GSE36807], Japanese [GSE233973], and Hungarian [GSE5081]) to enhance population diversity, this selection does not fully capture global heterogeneity. Genetic predispositions, environmental factors, and region‐specific pathogen strains or clinical practices may confound the expression signatures we observed, potentially affecting the generalizability of our diagnostic model to underrepresented populations. Finally, the use of bulk tissue RNA data limits our ability to deconvolute cell‐type‐specific contributions. The observed expression changes in immune‐related genes likely reflect a mixture of signals from various infiltrating immune cells and resident tissue cells. Future single‐cell RNA sequencing studies on co‐affected tissues could provide a higher resolution view of the cellular players driving these shared responses. Consequently, future research directions to overcome the above limitations should include: (1) Prospective validation in independent, multi‐ethnic large cohorts; (2) Functional studies of candidate hub genes using experimental models; (3) Integration of multi‐omics data (such as proteomics, metabolomics) to construct more comprehensive pathological networks. (4) Exploring the detailed molecular mechanisms underlying *H. pylori*‐induced tolerogenic dendritic cells and immunosuppressive regulatory T cells, and how these cell populations influence the onset and progression of UC. Such studies will help clarify whether *H. pylori* exerts protective effects in UC through the identified molecular hubs.

## 5 Conclusion

Our findings establish a molecular framework, emphasizing shared immune pathways in *H. pylori* infection and UC and identified six hub genes as key mediating factors. These insights not only connect gastric infection to colonic inflammation but also propose actionable targets for precision medicine in UC management.

## Author Contributions

**Min Zhu:** conceptualization, methodology, formal analysis, funding acquisition, writing – original draft. **Xiao Li:** conceptualization, methodology, formal analysis, funding acquisition, writing – original draft. **Xinyuan Huang:** formal analysis, data curation, visualization, writing – original draft. **Ruihua Dong:** conceptualization, methodology, data curation, funding acquisition, writing – review and editing. **Shutian Zhang:** conceptualization, methodology, data curation, writing – review and editing.

## Ethics Statement

This study did not require ethical committee approval as all patient data were retrieved from publicly accessible databases specified in the manuscript.

## Conflicts of Interest

The authors declare no conflicts of interest.

## Supporting information

Supplementary Table 1.

## Data Availability

All data generated or analysed during this study are included in this article and its supplementary information files.
